# A Case-Control Study for the Effectiveness of Oral Zinc in the Prevention and Mitigation of COVID-19

**DOI:** 10.3389/fmed.2021.756707

**Published:** 2021-12-13

**Authors:** Antonio M. Gordon, Patrick C. Hardigan

**Affiliations:** ^1^Department of Internal Medicine, University Health Care, Hialeah, FL, United States; ^2^Dr. Kiran C. Patel College of Allopathic Medicine, Nova Southeastern University, Fort Lauderdale, FL, United States

**Keywords:** COVID-19, zinc, morbidity and mortality, comorbidities, treatment

## Abstract

**Background:** The ongoing coronavirus disease-19 (COVID-19) pandemic (caused by an infection with severe acute respiratory syndrome (SARS)-coronavirus (CoV-2) has put a burden on the medical community and society at large. Efforts to reduce the disease burden and mortality over the course of the pandemic have focused on research to rapidly determine age-stratified seroepidemiologic surveys, a centralized research program to fast-track the most promising rapid diagnostics and serologic assays, and the testing of potential anti-viral agents, immunologic therapies, and vaccine candidates. Despite the lack of official recognition for the role of nutrition in the fight against COVID-19 infection, multiple groups proposed zinc supplementation as an adjuvant for the management of participants.

**Method:** In an ambulatory, interventional, prospective, single-blind study, we evaluated the effectiveness of zinc supplementation in the prevention and mitigation of COVID-19 in two similar participant groups. In Clinic A (*n* = 104) participants were randomized to receive 10 mg, 25 mg, or 50 mg zinc picolinate daily, and Clinic B control participants paired according to their demographics and clinical parameters (*n* = 96). All participants were compared based on demographics, clinical comorbidities, blood counts, renal functions, vitamin D levels, and their development of symptomatic COVID-19 infection.

**Results:** Symptomatic COVID-19 infection was significantly higher among the control group participants (*N* = 9, 10.4%) than the treatment participants (*N* = 2, 1.9%), *p* = 0.015. The unadjusted odds ratio indicates that symptomatic COVID-19 infection was 5.93 [95% CI: 1.51, 39.26] higher in the control group, *p* < 0.01. Controlling for co-morbidities, individuals in the control group were 7.38 (95% CI: 1.80, 50.28) times more likely to develop symptomatic COVID-19 infection as compared with individuals in the treatment group (*p* < 0.01). For every-one unit increase in the number of co-morbidities, the likelihood of developing symptomatic COVID-19 infection increased 1.57 (95% CI: 1.16, 2.19) (*p* = 0.01).

**Discussion:** The findings from our study suggest that zinc supplementation in all three doses (10, 25, and 50 mg) may be an effective prophylaxis of symptomatic COVID-19 and may mitigate the severity of COVID-19 infection.

**Conclusion:** Zinc is a relatively inexpensive mineral nutrient that is an effective prophylactic agent to prevent and mitigate the potentially deadly symptomatic SARS-CoV-2 infection. As the COVID-19 pandemic continues with a lag in vaccinations in some regions and the continued emergence of dangerously infectious variants of SARS-CoV-2, it is important to replicate our data in other populations and locations and to engage public health and nutrition services on the emergent need to use zinc supplantation or fortification of staple foods in the prevention and mitigation of COVID-19 infection severity.

## Introduction

The ongoing coronavirus disease-19 (COVID-19) pandemic (caused by an infection with severe acute respiratory syndrome (SARS)-coronavirus (CoV)-2) has put a strain on the medical community and society at large, as it has spread at a rapid pace globally (1). The pandemic has progressed since it was first identified as an epidemic of a new illness and manifested initially as pneumonia secondary to a novel coronavirus in December 2019 in Wuhan, China (2).

The infection ranges symptomatically among individuals from asymptomatic to milder symptomatology that includes fever, sore throat, cough, lung infections, and in more severe manifestations of the illness, acute respiratory distress syndrome (ARDS), sepsis, and death. The most reported co-morbidities including hypertension, diabetes, chronic obstructive pulmonary disease (COPD), obesity, and cardiovascular diseases, have been shown to put participants with COVID-19 infection at greater risk of severe adverse events and death (3). As many as 50 percent of participants have been reported to have at least one of these co-morbidities when admitted to hospital with COVID-19 infection.

Over the course of 2020 and into 2021, the World Health Organization (WHO) and the Centers for Disease Control and Prevention (CDC) aimed to prioritize research to rapidly determine age-stratified seroepidemiologic surveys, establish a centralized research program to fast-track the most promising rapid diagnostics and serologic assays, and the testing of potential anti-viral agents, immunologic therapies, and vaccine candidates (1). Despite the lack of official recognition for the role of nutrition in the prevention and mitigation of COVID-19 infection, multiple research and clinical groups proposed zinc supplementation as an adjuvant for the management of participants (4).

During the previous coronavirus pandemic of SARS and its aftermath, zinc supplementation was found to play an important role in the reproduction of SARS through its inhibition (*in vitro*) of the SARS CoV-1 RNA (5, 6). Furthermore, during the current COVID-19 pandemic, zinc has been identified as a clinical marker whose deficiency manifested clinically as hypozincemia and was strongly associated with serious complications including ARDS and increased mortality (4). Some researchers proposed to study the role of zinc supplementation in the prophylaxis of COVID-19 infection, but data has not been published on its effectiveness (7). Although the availability of effective vaccines against COVID-19 infection have started to ease the devastating effects of SARS-CoV-2 in some regions of the developed world, socio-economic and political issues and the lack of public health and technological infrastructure in most of the world have yet to resolve the ongoing pandemic, which is negatively impacting Latin America, the Caribbean, Asia, and Africa due to limited access to vaccination (2, 8, 9). As such, in May 2020, we proposed to evaluate the prophylactic effects of oral supplementation with zinc in out-patients in a community where SARS CoV-2 was circulating and COVID-19 infection was prevalent at a level of least 100 cases per 100,000 population.

## Methods

**Figure 1:**
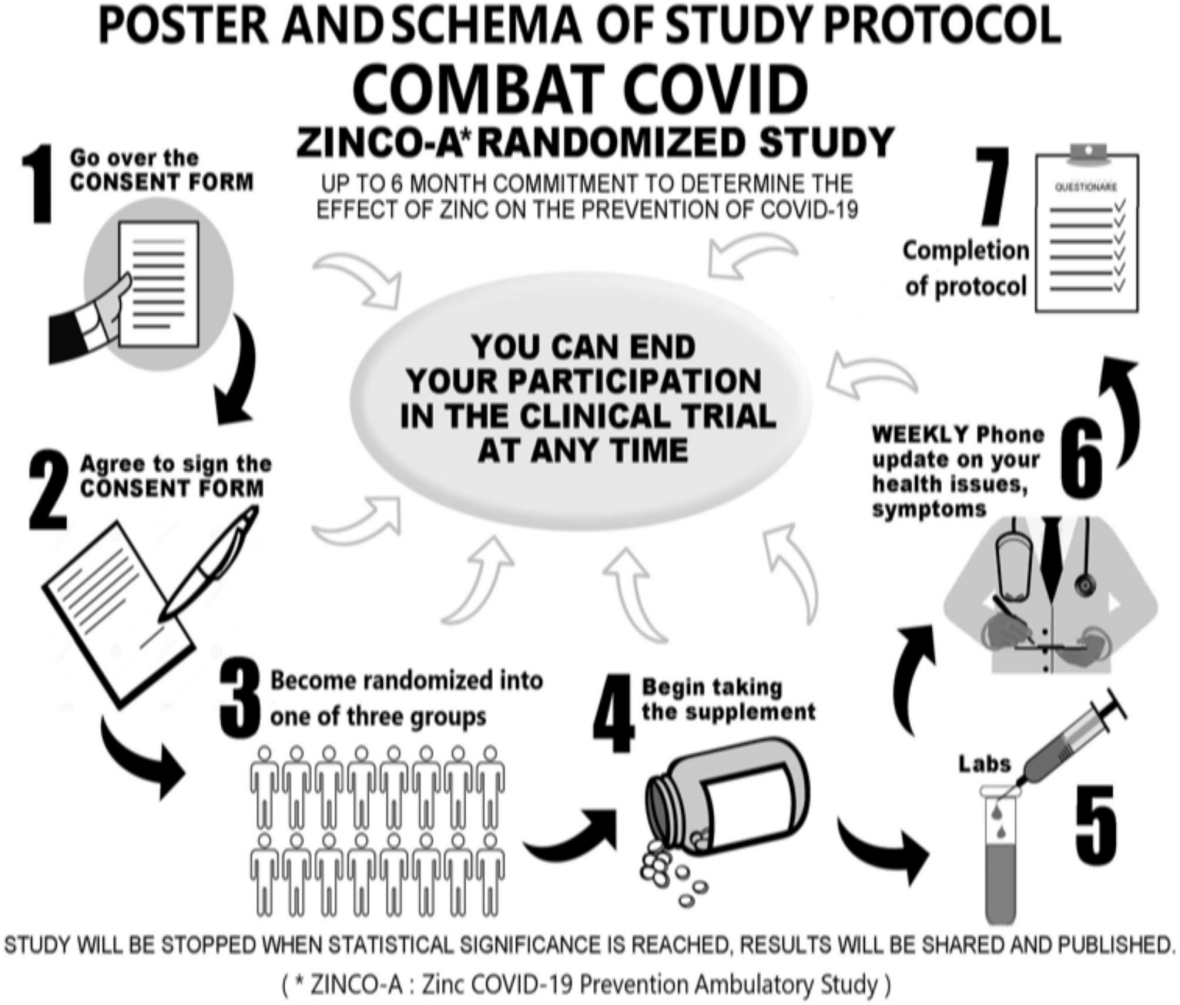
Promotional flier.

Participants enrolled in University Health Care (UHC) ambulatory primary care centers in Hialeah, Florida (FL) were recruited for the study group to be treated prophylactically with orally administered zinc. We initially designed a double-blind type of study using placebo capsules identical to the zinc capsules for two sets of participants selected at random from the volunteers who accepted our announcement. However, most participants in the internal medicine clinic were already taking a zinc supplement on the advice of their attending physician, AG. It was unethical to ask participants to stop taking zinc during the 2020 peaks of the pandemic at a time when the mineral supplement was already being promoted as being helpful against COVID-19 infection. Instead, we decided to study the dose of zinc necessary to prevent or mitigate COVID-19 infection from participants in AG's clinic, hereafter labeled Clinic A, who were willing to be randomized to one of three daily dose regimens: 10, 25, or 50 mg. A diagram was posted in the clinic visible to all participants attending the primary care UHC centers in Hialeah, FL illustrating the invitation to become participants in the COVID-19 prevention and mitigation clinical study (Figure 1). The controls would be participants from the adjacent UHC clinic (Clinic B) where zinc was not routinely used or recommended by their attending physicians. The study was open to all participants in the center, and a few of the clinic B participants participated in the randomized zinc dose treated group. Participants, 50–84 years of age, were recruited because individuals who acquired COVID-19 infection in this age range had the most likely emergence of complications leading to hospitalization and death (2). Exclusion criteria included taking zinc-containing supplements other than a multi-vitamin such as Centrum for adults, living outside the area of Miami-Dade and Broward counties, unable to follow instructions or dementia, taking immunosuppressive agents in the context of having had a transplant, diagnosis of rheumatoid arthritis or inflammatory arthritis, diagnosis of a terminal illness or hospice care, cirrhosis of the liver, hemodialysis being required on a chronic basis, human immunodeficiency virus disease, housebound persons, individuals known to have a history of intolerance to oral zinc supplements, and lastly having a positive test for having had or having SARSCoV-2 infection as demonstrated by a baseline laboratory test done in all Clinic A participants through a blood sample submitted to a clinical reference laboratory for SARS-CoV-2 IgG, IgM, or IgA antibodies in their serum.

**Figure 2:**
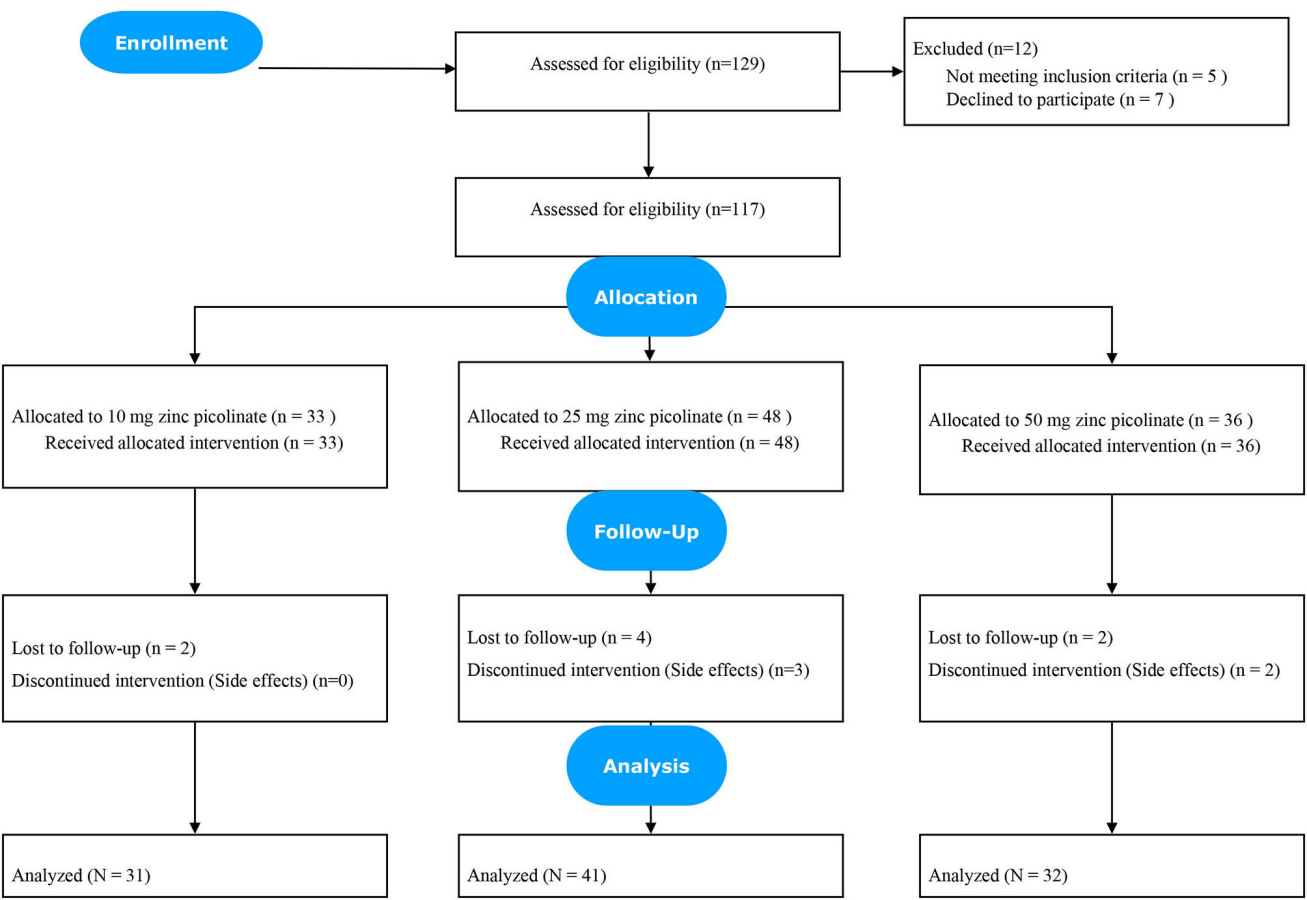
Clinic B: Flowchart of control participants.

Participants who were routinely seen in Clinic A who accepted to participate in the clinical study were randomized to receive 10, 25, or 50 mg oral zinc picolinate daily, specially compounded for this study from pharmaceutical grade product. Unrestricted randomization using a list of random assignments generated by a computer was employed, with supplement dosages masked to the participant. Participants were monitored by telemedicine every 2–3 weeks to collect data on the zinc supplement tolerance and monitor the symptoms of all participants enrolled in the study. All participants in the treated group were reminded during these telemedicine sessions not to take their zinc supplements with iron or calcium supplements. This guideline had been instructed at the outset to prevent issues with the intestinal absorption of zinc and was stressed in the inform consent document, which was reviewed and signed by all participants in the treated group. The process of refinement of the control participants is outlines in Figure 2.

**Figure 3:**
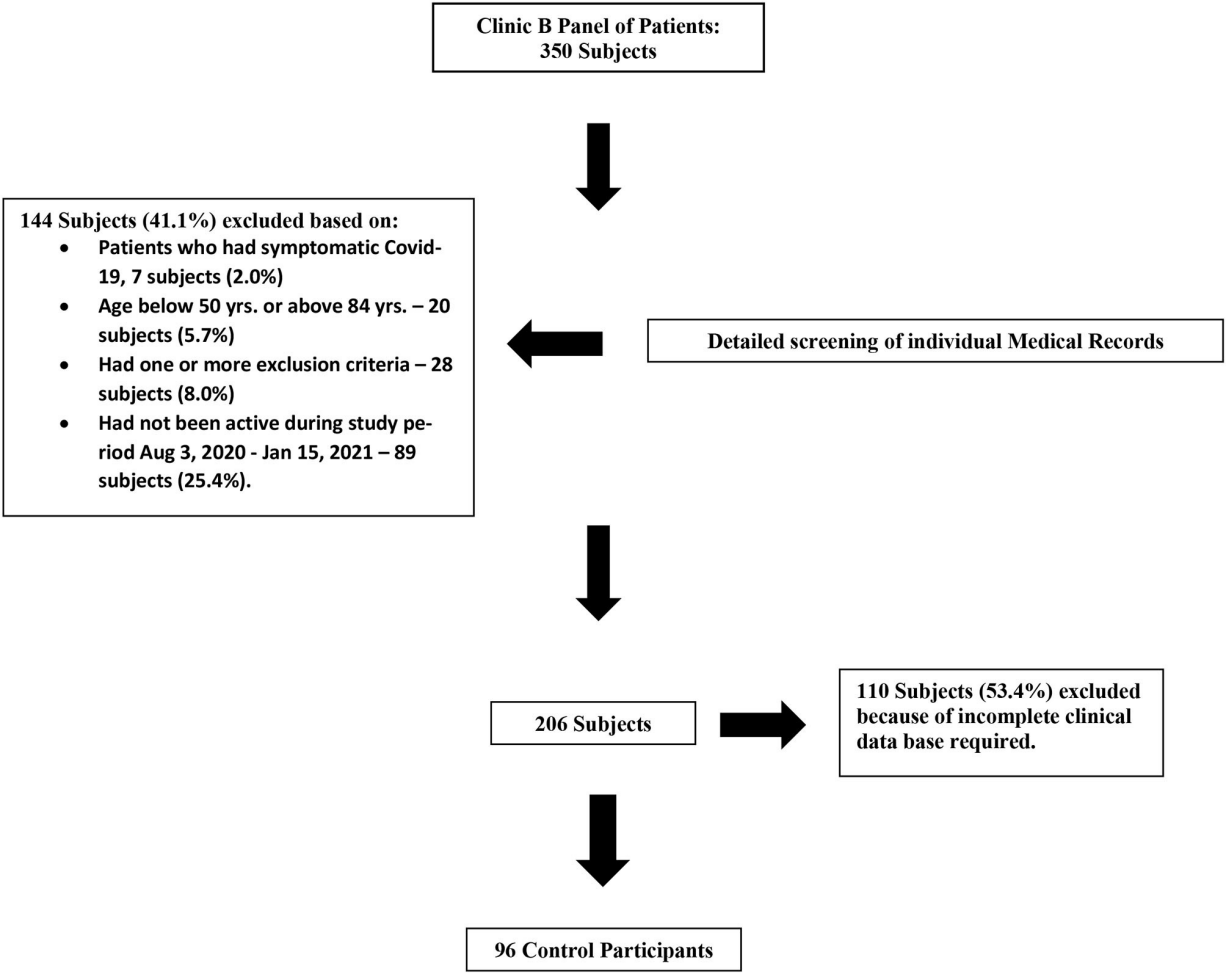
Clinic B: Flowchart control participants.

Permission was obtained from the medical attending in Clinic B and in January, 2021, we reviewed the electronic health records (HER) of their panel of participants from which we found 206 participants who had been active, seen a physician and had blood work done in the last 6 months and had none of the exclusion criteria noted above for Clinic A except for the presence or absence of SARS-CoV-2 antibodies in serum because Clinic B participants were not routinely assessed for these tests. The process of refinement of the control participants is outlines in Figure 3. Participants from Clinic B outside the ages 50–84 years or with any of the exclusion criteria listed above for Clinic A participants were discarded without further consideration. From the 206 controls obtained in this manner, 96 participants were further selected on their demographics and laboratory studies to be paired with the Clinic A recruited participants in the study. All participants from Clinic A and Clinic B had routine visits to the clinic during the study that involved participation in a COVID-19 questionnaire about the presence or absence of symptoms suggestive of COVID-19 infection and checked for compliance on precautions to avoid contagion with regards to physical distancing and appropriate use of antimicrobial gel and face masks. Baseline zinc levels were not available for participants from Clinic B. Both Clinic A and Clinic B participants share the same front desk at our Hialeah centers where the COVID-19 questionnaires were and are routinely administered to all participants during the pandemic when they were equally monitored and reminded of all recommended measures to avoid the COVID-19 contagion.

### Sample Size

We tested a superiority hypothesis or the use of Zinc in an ambulatory setting will results in a 20% or greater percentage of non-infected COVID-19 patients. All subject assessments will be measured by improvement of scores from baseline to end of treatment.

#### Primary Endpoints

Part A:

Ho: H0 = πc – πt = 10%
Ha: Ha = πc – πt ≥ 10%
Where
πc are the percentage of non-infected patients for the control group
πt are the percentage of non-infected patients for the treatment group.

We operationalized the percentage of symptomatic Covid-19 for the treatment vs. the control group as a dichotomous outcome variable (symptomatic yes vs. symptomatic no). The sample-size estimate was based on exact tests with actual levels of significance and power. In a two-arm study with P_0_ (unacceptable response rate) = 10%, P_1_ (response rate that is desirable) = 90%, specified α = 5% and power = 80%, the A'Hern approach yielded a minimum of *n* = 30 per arm.

### Statistical Methods

After the data were cleaned, we examined the distribution and dispersion of data through descriptive numerical summaries and graphical tools. Bivariate analysis using either a Fisher's Exact test or a Wilcoxon test was conducted between the independent variable group (treatment vs. control) and the dependent variables symptomatic Covid-19 (yes vs. no), race (white vs. other), gender, age (yrs.), body mass index (BMI, kg/m2), cardiovascular disease (yes vs. no), diabetes (yes vs. no), chronic obstructive lung disease [COPD] (yes vs. no), chronic renal failure (yes vs. no), recent cancer (yes vs. no), alcohol use (yes vs. no), coffee use (yes vs. no), tobacco use (yes vs. no), white blood cell count (WBC, 1000/mcL), hemoglobin (Hgb, g/dL), platelet count (1000/mcL), HgbA1c (%), creatin (cr, mg/dL), blood urea nitrogen (BUN, mg/dL), estimated glomerular filtration rate (eGFR), albumin (Alb, g/dL), vitamin D levels (ng/mL), and the number of co-morbidities. Logistic regression modeling was employed to look for associations between the dependent variable, symptomatic COVID (yes vs. no), the independent variable group (treatment vs. control) and other study variables. Model fit was tested using AIC, and to see if the data met the assumption of collinearity, we calculated VIF for each predictor. Mean values are presented with corresponding standard deviations. All hypothesis testing was carried out at the 5 percent (two-sided) significance level *a priori*. All subsequent statistical tests were conducted *post-hoc*. Missing data was deleted in all statistical analysis. *P*-values were rounded to three decimal places. *P*-values <0.001 were reported as <0.001 in tables. *P*-values >0.999 were reported as >0.999. R (version 4.2.0) statistical package was used for descriptive calculations, group comparisons, and regression modeling.

## Results

All 129 participants who initially agreed to participate in the study received their allotted bottle of zinc supplements within 5 days of signing up. Initial laboratory studies were not available until days later, at which time five participants were informed of having had asymptomatic SARS-CoV-2 infection and thus removed from further analysis. Within 1 week of having been accepted into the study, seven participants signed out from the study because of fears and concerns raised by their immediate family and children after they read the informed consent document previously signed by the participants. One hundred seventeen participants were then officially followed post-randomization. During the first 4 weeks of monitoring, five participants experienced intolerable side effects; two had headaches potentially attributable to zinc supplementation (one on 25 mg, and the other on 50 mg zinc picolinate), three participants experienced gastrointestinal symptoms including nausea and constipation and refused to take the zinc supplement (two participants on 25 mg and one participant on 50 mg zinc picolinate). One hundred twelve participants were available for analysis after the study).

Participants from Clinic A treated with oral zinc picolinate and control participants from Clinic B were statistically equivalent in their demographics, as well as in their prevalence of obesity, diabetes mellitus, and hypertension (Table 1). Both groups of participants resided in Miami-Dade and Broward counties in Southern Florida and were followed for their primary care in one of the two managed care ambulatory medical centers of UHC. Most of the participants in our study were Hispanic, as the geographic area where they reside has a population of 95 percent Hispanic. As noted earlier, a double-blind study could not be performed for ethical reasons because most participants in Clinic A had been on zinc supplementation and taking them off their zinc supplement was not appropriate or acceptable.

**Table 1:** Bivariate categorical comparisons.

	**Treatment**	**Control**	**Overall**	
	**(*N* = 104)**	**(*N* = 96)**	**(*N* = 200)**	***P*-value**
Gender				0.999
Female	64 (61.5%)	59 (61.5%)	123 (61.5%)	
Male	40 (38.5%)	37 (38.5%)	77 (38.5%)	
Cardiovascular disease				0.999
No	13 (12.5%)	3 (3.1%)	16 (8.0%)	
Yes	91 (87.5%)	93 (96.9%)	184 (92.0%)	
Diabetes				0.251
No	66 (63.5%)	53 (55.2%)	119 (59.5%)	
Yes	38 (36.5%)	43 (44.8%)	81 (40.5%)	
COPD				0.033
No	49 (47.1%)	60 (62.5%)	109 (54.5%)	
Yes	55 (52.9%)	36 (37.5%)	91 (45.5%)	
Chronic renal failure				0.069
No	84 (80.8%)	87 (90.6%)	171 (85.5%)	
Yes	20 (19.2%)	9 (9.4%)	29 (14.5%)	
Recent cancer				0.825
No	93 (89.4%)	84 (87.5%)	177 (88.5%)	
Yes	11 (10.6%)	12 (12.5%)	23 (11.5%)	
Alcohol				0.347
No	78 (75.0%)	66 (68.8%)	144 (72.0%)	
Yes	26 (25.0%)	30 (31.2%)	56 (28.0%)	
Coffee				0.557
No	14 (13.5%)	16 (16.7%)	30 (15.0%)	
Yes	90 (86.5%)	80 (83.3%)	170 (85.0%)	
Tobacco				0.448
No	97 (93.3%)	86 (89.6%)	183 (91.5%)	
Yes	7 (6.7%)	10 (10.4%)	17 (8.5%)	
Symptomatic Covid-19				0.015
No	102 (98.1%)	86 (89.6%)	188 (94.0%)	
Yes	2 (1.9%)	10 (10.4%)	12 (6.0%)	
Covid mortality				0.480
No	104 (100%)	95 (99.0%)	199 (99.5%)	
Yes	0 (0%)	1 (1.0%)	1 (0.5%)	

A total of 200 participants were enrolled in the study (treatment = 104, control = 96). Overall, the median age was 74 (IQR: 69–79), 90 percent were Hispanic white, 62 percent were female, and the median BMI was 28.50 (IQR: 25.57–32.15). Participants in the study from Clinic A had 3.47 ± 0.83 encounters during the time of the study from August 2020 until January 2021. Participants from Clinic B whose records were selected as controls for the study had 3.08 +/−1.60 encounters where they were equally monitored and reminded of all recommended measures to avoid the COVID-19 contagion. Treated and control participants also had statistically equivalent hematologic and clinical chemistry parameters (Table 2). In addition, the participants who were paired from Clinic B (*n* = 96) had a vitamin D serum level comparable from the treated participants. All participants in Clinic A had a pre-study and a post-study laboratory test for SARS-CoV-2 IgG antibodies. Two participants from Clinic A also had a positive SARS-CoV-2 IgG antibody test corresponding to those two individuals who had symptomatic COVID-19 infection.

**Table 2:** Bivariate continuous comparisons.

	**Treatment**	**Control**	**Overall**	***P*-value**
	**(*N* = 104)**	**(*N* = 96)**	**(*N* = 200)**	
Age				0.003
Mean (SD)	74.0 (6.74)	71.1 (8.15)	73.8 (6.83)	
Median [Min, Max]	74.0 [50.0, 85.0]	71.0 [52.0, 82.0]	74.0 [50.0, 85.0]	
Missing	1 (0.5%)	0 (0%)	1 (0.5%)	
BMI				0.127
Mean (SD)	29.1 (5.03)	31.8 (4.09)	29.3 (5.02)	
Median [Min, Max]	28.7 [16.6, 46.7]	32.1 [25.1, 37.2]	28.9 [16.6, 46.7]	
WBC				0.452
Mean (SD)	7.20 (2.24)	6.41 (1.79)	7.16 (2.23)	
Median [Min, Max]	6.80 [2.00, 13.6]	5.55 [4.70, 10.1]	6.70 [2.00, 13.6]	
Missing	12 (6.2%)	1 (9.1%)	13 (6.3%)	
Hgb				0.907
Mean (SD)	20.6 (95.9)	12.9 (1.43)	20.2 (93.4)	
Median [Min, Max]	13.4 [6.80, 1310]	12.5 [11.5, 15.7]	13.4 [6.80, 1310]	
Missing	12 (6.2%)	1 (9.1%)	13 (6.3%)	
Platelet count				0.686
Mean (SD)	243 (66.4)	252 (70.4)	243 (66.5)	
Median [Min, Max]	237 [93.0, 447]	237 [174, 411]	237 [93.0, 447]	
Missing	13 (6.7%)	1 (9.1%)	14 (6.8%)	
HgbA1c				0.617
Mean (SD)	7.25 (1.20)	7.15 (1.24)	7.24 (1.19)	
Median [Min, Max]	7.00 [5.40, 9.70]	7.20 [5.50, 8.70]	7.00 [5.40, 9.70]	
Missing	132 (68.0%)	5 (45.5%)	137 (66.8%)	
Cr				0.936
Mean (SD)	1.49 (6.92)	1.04 (0.333)	1.46 (6.74)	
Median [Min, Max]	0.870 [0.520, 94.0]	0.985 [0.630, 1.50]	0.870 [0.520, 94.0]	
Missing	12 (6.2%)	1 (9.1%)	13 (6.3%)	
Bun				0.471
Mean (SD)	18.2 (5.43)	20.9 (5.57)	18.3 (5.46)	
Median [Min, Max]	18.0 [8.00, 38.0]	21.0 [13.0, 30.0]	18.0 [8.00, 38.0]	
Missing	12 (6.2%)	1 (9.1%)	13 (6.3%)	
eGRF				0.563
Mean (SD)	73.4 (19.5)	67.4 (19.1)	73.1 (19.5)	
Median [Min, Max]	76.0 [24.0, 158]	68.5 [44.0, 93.0]	75.5 [24.0, 158]	
Missing	12 (6.2%)	1 (9.1%)	13 (6.3%)	
Alb				0.444
Mean (SD)	4.44 (0.278)	4.39 (0.370)	4.43 (0.282)	
Median [Min, Max]	4.40 [3.50, 5.20]	4.30 [3.90, 5.10]	4.40 [3.50, 5.20]	
Missing	15 (7.7%)	1 (9.1%)	16 (7.8%)	
Vitamin D				0.559
Mean (SD)	41.3 (15.4)	34.4 (14.3)	40.9 (15.4)	
Median [Min, Max]	39.0 [14.2, 98.0]	33.1 [21.0, 69.6]	38.6 [14.2, 98.0]	
Missing	14 (7.2%)	1 (9.1%)	15 (7.3%)	
Comorbidities^*^				0.458
Mean (SD)	5.27 (2.25)	5.05 (2.07)	5.17 (2.17)	
Median [Min, Max]	5.00 [1.00, 9.00]	5.00 [1.00, 9.00]	5.00 [1.00, 9.00]	

* *Comorbidities refers to cardiovascular disease, diabetes, COPD, chronic renal failure, recent cancer (<2 years), smoking and alcohol use*.

Fisher's exact test revealed that the experimental group 1.92% (*N* = 2) had significantly fewer symptomatic COVID-19 infections than the control group 10.42% (*N* = 10) (95% CI: 1.20, 56.61) (*p* = 0.01). Statistical analysis between the experimental and control also revealed group differences for the variables age and chronic obstructive lung disease–Tables 1, 2 (*p* < 0.05). Specifically, the treatment group was older (Median difference = 3 years, [95% CI: 1.26, 4.00, *p* = 0.003]), and had more COPD (Percentage difference = 15.41%, [95% CI: 0.09,29.20, *p* = 0.033]). No difference in COVID-19 symptomology was found between the different dosage groups. No participant receiving 10 mg of zinc developed COVID-19 symptoms; meanwhile, one participant each in the 25 mg and 50 mg zinc picolinate daily groups reported COVID-19 symptoms.

Of the two participants who developed COVID-19 infection, they had mild symptoms–cough, sore throat, low-grade fever, and generalized malaise. Both participants were managed in the outpatient service via telemedicine without complications. In the control group, there were nine cases of symptomatic COVID-19 infection, and three participants required hospital admission for severe hypoxemia, and one of these hospitalized participants died.

Based on clinical judgment and the statistical results, we created six logistic regression models. Model fit was tested using the Akaike information criterion (AIC) and likelihood ratio tests (LR). To ascertain if the models met the assumption of collinearity, we calculated a variance inflation factor (VIF) for each predictor. For our study, a VIF of 1 indicated no correlation among the kth predictor and the remaining predictor variables. The general rule of thumb is that VIFs exceeding 4 warrant further investigation, while VIFs exceeding 10 are signs of serious multicollinearity requiring correction.

- Base Model: Experimental group
- Model 1: Experimental group + number of comorbidities
- Model 2: Experimental group + number of comorbidities + age
- Model 3: Experimental group + number of comorbidities + age + BMI
- Model 4: Experimental group + number of comorbidities + age + BMI + COPD
- Model 5: Experimental group + number of comorbidities + age + BMI + COPD + gender + vitamin D levels.

AIC values increased by model (adding additional predictors); however, LR tests indicted no significant difference between the models. Meaning no significant reduction in residual deviance and higher AIC values. No VIF was larger than our threshold of 4, in fact, all were close to one.

**Figure 4:**
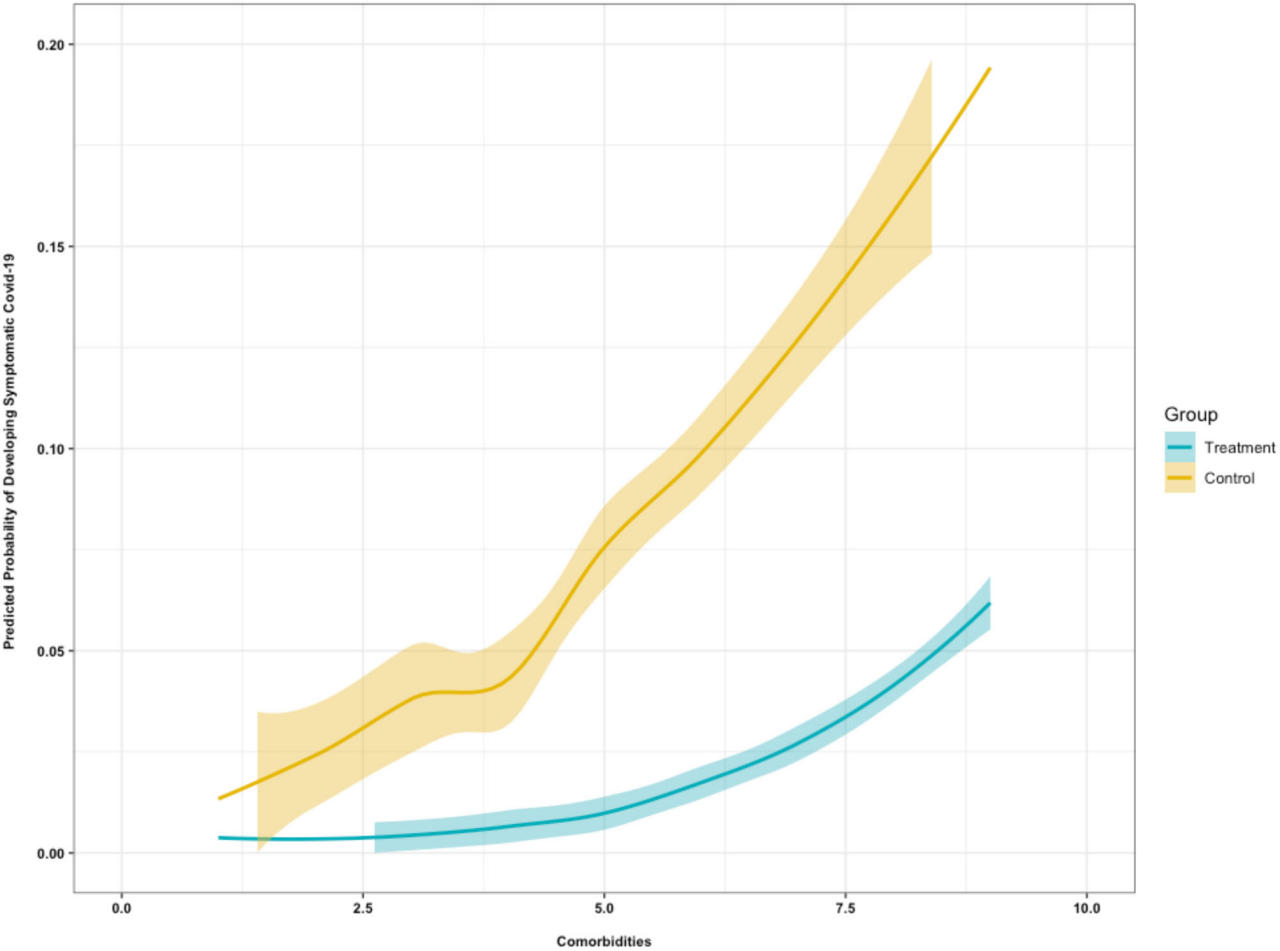
Predicted probability of developing symptomatic COVID-19 based on comorbidities.

Results from the logistic regression models–Table 3 and Figure 4–revealed.

- Base Model: Individuals in the control group were 5.93 (95% CI: 1.51, 39.26) times more likely to develop symptomatic COVID-19 infection as compared with individuals in the treatment group (*p* = 0.01).
- Model 1: Controlling for co-morbidities, individuals in the control group were 7.38 (95% CI: 1.80, 50.28) times more likely to develop symptomatic COVID-19 infection as compared with individuals in the treatment group (*p* < 0.01). For every-one unit increase in the number of co-morbidities, the likelihood of developing symptomatic COVID-19 infection increased 1.57 (95% CI: 1.16, 2.19) (*p* = 0.01).
- Model 2: Controlling for comorbidities and age, individuals in the control group were 6.89 (95% CI: 1.67, 47.08) times more likely to develop symptomatic COVID-19 infection as compared with individuals in the treatment group (*p* = 0.01). For every-one unit increase in the number of co-morbidities, the likelihood of developing symptomatic COVID-19 infection increased 1.60 (95% CI: 1.20, 2.38) (*p* = 0.01).
- Model 3: Controlling for comorbidities, age, and BMI, individuals in the control group were 6.92 (95% CI: 1.68, 47.39) times more likely to develop symptomatic COVID-19 infection as compared with individuals in the treatment group (*p* = 0.02). For every-one unit increase in the number of co-morbidities, the likelihood of developing symptomatic COVID-19 infection increased 1.64 (95% CI: 1.19, 2.36) (*p* < 0.01).
- Model 4: Controlling for comorbidities, age, BMI, and COPD, individuals in the control group were 6.31 (95% CI: 1.50, 43.55) times more likely to develop symptomatic COVID-19 infection as compared with the treatment group (*p* = 0.02). For every-one unit increase in the number of co-morbidities, the likelihood of developing symptomatic COVID-19 infection increased 1.67 (95% CI: 1.22, 2.42; *p* < 0.01).
- Model 5: Controlling for comorbidities, age, BMI, COPD, gender and vitamin D levels, individuals in the control group were 6.09 (95% CI: 1.45, 42.12) times more likely to develop symptomatic COVID-19 infection as compared with the treatment group (*p* = 0.03). For every-one unit increase in the number of co-morbidities, the likelihood of developing symptomatic COVID-19 infection increased 1.68 (95% CI: 1.22, 2.43; *p* < 0.01).

**Table 3:** Logistic regression model results.

**Predictors**	**OR**	**CI**	**OR**	**CI**	**OR**	**CI**	**OR**	**CI**	**OR**	**CI**	**OR**	**CI**	**OR**	**CI**
(Intercept)	0.06	0.04–0.11	0.02	0.00–0.06	0.00	0.00–0.01	0.05	0.00–76.41	0.03	0.00–11.90	0.03	0.00–13.26	0.03	0.00–14.88
Treatment group														
Control group			5.93^*^	1.51–39.26	7.38^*^	1.81–50.29	6.89^*^	1.68– 7.09	6.93^*^	1.68– 7.39	6.32^*^	1.51– 3.56	6.09^*^	1.45–42.12
Comorbidities					1.57^***^	1.17–2.20	1.66^**^	1.21–2.38	1.64^**^	1.20–2.37	1.68^**^	1.22–.43	1.68^**^	1.22–2.43
Age							0.95	0.85–1.05	0.95	0.85–1.06	0.95	0.85–.06	0.95	0.85–1.06
BMI									1.02	0.90–1.15	1.02	0.90–1.15	1.01	0.89–1.15
CLD Yes														
CLD No										0.64	0.23–1.58	0.63	0.22–1.59	
Vit.D Levels														
													1.00	0.95–1.05
Female														
Male													1.05	0.28–8.69
N	200		200		200		200		200		200		197	
*R*^2^ Tjur	0.00		0.03		0.14		0.12		0.12		0.12		0.12	
Log likelihood	−45.39		−41.96		−37.39		−36.86		−36.81		−36.34		−36.00	
Akaike Inf. Crit.	92.79		87.92		80.77		81.73		83.61		84.69		87.99	

*OR refers to Odds Ratios. CI are 95% Confience Intervals*.

* *p < 0.05*;

** *p < 0.01*;

*** *p < 0.001*.

## Discussion

This study found that zinc supplement was associated with a reduced risk in developing symptomatic COVID-19. Controlling for co-morbidities, individuals in the treatment group were also less likely to develop symptomatic COVID-19 infection as compared with individuals in the control group. Additionally, it was found that co-morbidities, regardless of groups, increased the likelihood of developing symptomatic COVID-19 infection. The findings from our study suggest that zinc supplementation may be an effective prophylaxis of symptomatic COVID-19 and may mitigate the severity of COVID-19 infection.

It is important to note that these data should not be interpreted to support the use of oral zinc in participants with symptomatic COVID-19 infection admitted to the hospital or the ICU. The latter will likely need close monitoring of their zinc status and appropriate intravenous supplementation (10). We and others have used intravenous zinc in the past in participants with HIV disease successfully (11, 12).

COVID-19 is a disease with a wide clinical spectrum that ranges from asymptomatic carriers to symptoms such as fever, sore throat, cough, lung infections, and in severe cases, ARDS, sepsis, and death (4). As many as 50 percent of participants admitted to hospital have been reported having at least one comorbidity, and ~70 percent of participants who require intensive care unit (ICU) care have been observed to have co-morbidities. The common comorbidities that have been identified that places participants at a greater propensity for serious complications and death have been obesity, diabetes, COPD, cardiovascular diseases including hypertension, malignancies, and human immunodeficiency virus disease (HIV). Participants with HIV were not included in our study because HIV disease is widely known to be associated with zinc deficiency and our participants with HIV are followed closely to keep their zinc nutritional status as optimal as possible.

In terms of demographics, an analysis of our patient population, revealed that the control participants were slightly younger than the zinc treated participants. Since SARS-CoV-2 tends to affect older individuals more severely, this minor difference in our two study groups places the zinc treated participants from Clinic A presumably at a greater risk of acquiring COVID-19 infection and suffering its serious adverse events or even death as compared with the paired control participants from Clinic B (Table 1). Still, the results revealed that after adjusting for age the observed protective effects from zinc picolonate supplementation remained significant. This is contrary to what may have been expected if the effect of zinc supplementation was not appreciably in favor of the treated group. Participants treated with zinc prophylactically faired significantly better statistically than those who did not take zinc-Table 1.

Vitamin D has been recognized as an important immune system active agent whose deficiency is associated with acquiring SARS-CoV-2 and serious or fatal outcomes from COVID-19 (12). In the paring of participants from Clinic B to our treated participants from Clinic A, we used not only the exclusion criteria noted above for the study but also the vitamin D status of participants. We were fortunate to find that Clinic B participants had their vitamin D levels updated regularly. Unfortunately, none of the Clinic B participants had ever had their zinc levels measured because it is not a laboratory test routinely conducted in primary care. However, the deleterious effects of vitamin D deficiency on participants with COVID-19 have been known and demonstrated to be an important factor associated with greater risk of hospitalization and mortality, particularly in Hispanic participants (8). Having paired our controls to our treated participants for vitamin D status allows us to concentrate on the role of zinc in the prevention of SARS-CoV2 infection in our mostly Hispanic population.

When controls and treated participants were compared by gender and history of cardiovascular disease (CVD), both groups were essentially equal. The presence of diabetes in both groups was slightly higher in the control group (44.8 percent) as compared with the treated group (36.5%). This is an area where the control group would have been expected to do worse than the treated group. Meanwhile, the HgbA1c level in both groups was similar at 7.16 percent in the treated group and 7.11 percent in the control group.

Chronic kidney disease (CKD) III or IV was present in 19.2 percent of the treated participants and 9.4 percent of the controls. This parameter would also place the treated participants at greater theoretical odds of having COVID-19 infection as compared with the control group. In addition, when both groups were compared in terms of their laboratory determinations for serum creatinine, both groups were fairly similar. This suggests that the CKD burden in the treated group was larger than in the control participants; however, the results demonstrate that the treated participants faired statistically better than the controls despite the increased burden of this illness. It should also be discussed here that the fact that the renal functions of both groups were found to be not significantly different suggests that both clinics under independently practicing physicians had coded properly the issue of CKD in their participants. However, Clinic A had more CKD III and IV, not on hemodialysis, than Clinic B.

In terms of chronic obstructive lung disease (COPD), the treated and control groups were populated by 52.9 percent and 37.5 percent of individuals with COPD. When all of these factors and others summarized in Table 1 were compared, we concluded that both groups were essentially similar, with the caveat that older age, CKD, and COPD were more prevalent among the treated participants, which should have placed them at a greater theoretical risk of developing COVID-19 infection and making them also theoretically more prone to COVID-19 infection complications and at increased mortality risk. The one area where the controls had a slightly higher, but not statistically significant number of individuals at a greater theoretical risk for COVID-19 was in the area of diabetes mellitus. Still, the HgA1c levels of both groups were essentially equivalent. Therefore, both treated and control groups can be declared similar but with a propensity of the treated group to be at a slightly higher risk of COVID-19 infection. This very fine difference between the groups gives the results of the study a greater significance, as the treated group did appreciably and statistically better than the control group in terms of their protection against COVID-19 infection and its complications.

In the developed world, it may be useful at this time to offer those individuals who for whatever reason have refused to undergo vaccination against COVID-19 and recommended to them to consider taking in a disciplined manner a supplement of 10 mg or 25 mg zinc daily. Most participants should be able to tolerate well 10 mg daily zinc picolinate, but without specific data, we feel those participants with co-morbidities known to be associated with severe COVID-19 infection or increased risk of mortality to use 25 mg daily. Further data on this recommendation is needed. It is felt that in each of these settings, individuals will be well-served in terms of their protection against the dangerous contagion when they are prophylactically suspended with oral zinc supplementation. Perhaps for some individuals who also refuse to take an oral supplement daily, some modification of their diet to include sufficient zinc to at least meet the RDA at 11 mg for men and 8 mg for women should be considered strongly.

Relatedly, we have witnessed lower vaccine efficacy against SARS-CoV-2 variants. Initial research showed that dose vaccine effectiveness against the alpha variant for hospitalization was 69.4% for the BNT162b2 (Pfizer) vaccine and 76.7% for the ChAdOx1 nCoV-19 (Oxford–AstraZeneca) vaccine (13). Recent research on the effectiveness after one dose of the Pfizer vaccine showed it was lower among persons with the delta variant-−30.7%. With the Oxford-AstraZenca vaccine, the effectiveness after one dose was 48.7% (14). Zinc supplements deserve consideration here as well because of additional benefits they provide for upper respiratory tract infections (15).

All participants in Clinic A had a pre-study and a post-study laboratory blood test for SARS-CoV-2 IgG antibodies. We found that no participants in Clinic A on zinc supplements had asymptomatic COVID-19 infection. None of the 104 participants in the treated group who were asymptomatic had a positive test for SARS-CoV2 IgG antibodies in their serum. The two individuals who did have a positive SARS-CoV-2 IgG antibody were the two who had symptomatic COVID-19 infection, and were treated in the outpatient service after they tested positive for SARS-CoV-2 antigen via a nasal swab test in the community. This has a very valuable epidemiologic perspective since, if our findings are replicated at other locations and in additional populations, oral zinc supplementation even at 10 mg daily may be beneficial in the prevention of the spread of the contagion from asymptomatic individuals to susceptible hosts during the COVID-19 pandemic.

SARS-CoV-2 has been known to affect the immune response resulting from its presence in the human body (16). It is important to highlight that the role of zinc in the human immune response encompasses activity in the innate and acquired immune responses, cell-mediated immunity, and also humoral B-cell mediated immunity (17).

Participants who are severely zinc-deficient suffer from lymphopenia, a decreased CD4/CD8 ratio, decreased natural killer (NK) cell activity, and increased monocytic cytotoxicity (18, 19). Correction of zinc nutritional status resolves all these abnormalities in the human immune response. Our study was not designed to determine the precise mechanism of action through which zinc affords prevention to our participants. However, because all treatment groups, 10 mg, 25 mg, and 50 mg, seemed to fare equally well in terms of protection against SARS-CoV-2 when compared with untreated participants, it is possible to speculate that the effect through which zinc helped our treated participants did not require to fully replenish their zinc nutritional status (18, 19). Along this line of reasoning, zinc can be thought of as being able to be a modulator of immune functions, a modifier of the interaction of SARS- CoV-2 with a host factor such as an angiotensin-converting enzyme (ACE)-2 receptor, or the integrity of the epidermal tight junctions, or even an agent capable of acting in the microbiome. These three modalities of action of zinc may be invoked as possible sites where the mineral exerts its protective effect in terms of the prevention of SARS-CoV-2 infection. Furthermore, zinc has been known to serve as a modulator of various immune functions including T cell interactions and functions and cytokine production.

However, with regards to the cytokine response to SARS-CoV-2, it has been proposed that a cytokine release, or also referred to as “cytokine storm,” is the pathophysiologic mechanism underlying the progression of COVID-19 infection from a relatively benign to a severe illness, which can result in significant morbidity and mortality. Therefore, the role of zinc in the therapeutics of COVID-19 is not addressed in this study but needs to be systemically approached to abate the morbidity and improved the mortality of these participants. In this context, it has been postulated that zinc could be a needed factor for the proper cytokine functions. This hypothesis applies more to the use of zinc in the therapeutics of COVID-19 infection, but the replenishment of zinc would be intuitively more difficult to achieve if a deficiency exists, to begin with.

The antiviral properties of zinc are virus-specific, but it appears that zinc ion availability plays a significant role in the antiviral efficacy of zinc (15). Additionally, the literature shows the importance of Zn as an essential mineral immunomodulator with relevant antiviral activity in the body (20). In terms of the ACE-2 receptor, it has been known that SARS-CoV-2 utilizes ACE-2 receptors found at the surface of the host cells to get inside the host cell. ACE-2 is a zinc metalloenzyme and in certain comorbidities are associated with a strong ACE-2 receptor expression and higher release of proprotein convertase that enhances the viral entry into the host cells (6). Meanwhile, zinc ions are capable of downregulating the response (21). As a result, it is plausible to suggest that zinc supplementation in relatively low doses may protect against COVID-19 infection by the downregulation of the ACE-2 enzyme and receptor through, which SARS-CoV-2 gains entry into our patient's host cells. In addition, zinc is active within the microbiome and gastrointestinal tract. Therefore, it can be hypothesized that the prevention of symptomatic infection with SARS-CoV-2 may be mediated through this pathway (22). The precise mechanism involved will require further specific studies in all of these complex areas of zinc physiology and SARS-CoV-2 pathophysiology.

Zinc deficiency is not uncommon since according to HANES III from 35 to 45% of American adults 60 years or older have a zinc deficiency (23). It has been proposed that the higher mortality associated with COVID-19 infection among elderly participants may be related to undiagnosed zinc deficiency (21). Elderly participants are prone to zinc deficiency due to the presence of chronic diseases and potentially due to the consumption of zinc-deficient diets. The dietary intake of zinc is deficient among 52 percent of elderly men and 34 percent of elderly women (22). This is understood in the context for the treatment of COVID-19 infection. Recent research found that the zinc status of COVID-19 patients did not exhibit a significant role in development of anosmia and/or hyposmia or disease severity; nevertheless, zinc therapy may play a significant role in shortening the duration of smell recovery in patients without affecting the total recovery duration from COVID-19 (24, 25).

We have performed a study in our UHC Hialeah participants recently and found by looking at 24-h dietary recalls that women consume 7.01 +/- 2.61 mg Zn daily less than the zinc RDA for women (23).

Two out of three of the women consume a zinc-deficient diet. Men did not fare much better having 4 out of 5 individuals consuming less than the RDA for men (26). The men consumed 9.72 +/−5.9 mg Zn daily. The best sources of dietary zinc in the American diet are beef, crustaceans, some beans, poultry, and fortified cereals (9). It is known that ageusia and dysgeusia, symptoms that have been reported infrequently among COVID-19 participants, have been known to be associated with zinc deficiency for more than 30 years (23).

The public in general, at least in the United States, is not always well-informed on the role of zinc in their immune system functions. In addition, primary care providers are not generally well-versed in evaluating zinc deficiency and may be unsure about its management. Furthermore, zinc levels in the components of blood have not always correlated with a total scarcity of this trace mineral. Earlier concerns about variations in zinc levels in blood were better understood when it was found that serum zinc levels are 16 percent greater than plasma zinc levels (4). This is due to the release of intracellular zinc from erythrocytes and platelets in the process of clotting. In addition, the hour of sampling blood for zinc determinations should be the same days because there is a diurnal variation in the level of zinc in the blood (26). Plasma zinc levels can be 67 percent lower in the early evening when compared to the levels determined at 08:00 h (16, 27, 28). Some commercial laboratories make no distinction between plasma or serum zinc determinations. Furthermore, a rather wide range of “normal” plasma zinc levels are reported which are not in agreement with the recommendations from the nutrition literature suggesting a range from 80 mcg to 135 mcg in plasma for humans (4).

### Limitations

Our study has limitations. Most of the participants included in the study were Hispanic, and caution must be exercised in extrapolating our data to other ethnic groups without first attempting to duplicate our findings. Other limitations include no adata about control subjects' zinc level or vaccination status was collected and relevant comorbidities. In addition, all our participants receive primary care in a single medical ambulatory center. As such, a multi-center, double-blind study is warranted to validate our findings and determine if they can be extrapolated to other locations and populations. Currently, it remains ethical to withhold COVID-19 vaccination where it is available. Meanwhile, it may be possible in certain communities where COVID-19 vaccination is not widely available, to evaluate the effect of zinc supplementation in relatively low doses and follow unvaccinated participants for at least 6 months to determine if indeed the protection we have reported can be replicated. In addition, such a double-blind study may be proposed ethically in locations in Latin America, the Caribbean, Southern Africa, or Asia where the prospects for vaccination remained limited.

## Conclusion

Overall, zinc supplementation must be further studied as our data suggests that it can be used to protect populations that are now vulnerable to succumb to SARS-CoV-2, including those participants with multiple co-morbidities. Zinc deficiency is a co-morbidity that places participants infected with SARS-CoV-2 at greater statistical risk of having complications. Our data also suggest that individuals using zinc, even in low doses, were protected from developing asymptomatic COVID or acquiring COVID from close relatives who had been symptomatically affected by the virus. Furthermore, participants who took their zinc supplement daily and who did not develop relatively mild cases of COVID-19 did not have evidence in their blood of having had asymptomatic SARS-CoV-2 infection. Since it is now imperative to replicate our findings in other populations and locations, it is necessary to seek support to demonstrate the positive role of oral zinc supplementation in the prevention of COVID-19. assess the zinc nutritional status by, measuring the plasma zinc level when possible, and heed attention to the zinc content of dietary intake in various populations including Hispanics in other locations in the US, non-Hispanics, and African-American communities to improve our ability to control the COVID-19 pandemic. The effect of zinc supplementation should also be important for individual health care workers, school administrators, teachers, students, and participants in long-term care nursing facilities among others. These arguments are also important to heed in the battle against COVID-19 infection globally since the prophylactic use of zinc orally to prevent or mitigate COVID-19 symptomatology may assist individuals in countries where the vaccine has lagged behind Europe and North America.

## Data Availability Statement

The raw data supporting the conclusions of this article will be made available by the authors, without undue reservation.

## Ethics Statement

The studies involving human participants were reviewed and approved by Sal Castaner, JD and VP of University Health Care for legal issues and compliance. The patients/participants provided their written informed consent to participate in this study.

## Author Contributions

AG designed and implemented the study. PH performed the data analysis. All authors discussed the results and contributed to the final manuscript.

## Conflict of Interest

The authors declare that the research was conducted in the absence of any commercial or financial relationships that could be construed as a potential conflict of interest.

## Publisher's Note

All claims expressed in this article are solely those of the authors and do not necessarily represent those of their affiliated organizations, or those of the publisher, the editors and the reviewers. Any product that may be evaluated in this article, or claim that may be made by its manufacturer, is not guaranteed or endorsed by the publisher.
